# Lecture on Certain Diseases of the Brain and Nervous System, with Special Reference to the Changes in Opinion and Practice Which Result from Recent Researches in Physiology and Pathology

**Published:** 1863-10

**Authors:** Charles Bland Radcliff

**Affiliations:** Fellow of the Royal College of Physicians, Physician to Westminster Hospital, etc.


### LECTURE ON CERTAIN DISORDERS OF THE BRAIN AND NERVOUS SYSTEM,

WITH SPECIAL REFERENCE TO THE CHANGES IN OPINION AND PRACTICE WHICH RESULT FROM RECENT RESEARCHES IN PHYSIOLOGY & PATHOLOGY.

DELIVERED AT THE

ROYAL COLLEGE OF PHYSICIANS,

By CHARLES BLAND RADCLIFF, M.D., Fellow of the Royal College of Physicians, Physician to Westminster Hospital, etc.

### LECTURE V.—Part II.

### (3) ON THE PATHOLOGY OF CONVULSION AS DEDUCED FROM THE CONDITION OF THE INNERVATION IN THIS DISORDER.

Reprinted from the "London Lancet."

86. *The signs of a weak or jaded brain are scarcely ever absent in persons who are liable to epileptic and other chronic forms of convulsive disorders.*

It is an indisputable fact, that idiots are very often epileptic, and it is surely no mere accident that the head of the epileptic is so often wanting in proper dimensions and proportions as to suggest to the least imaginative observer its near kinship to the head of the idiot. It is also an indisputable fact, that a not uncommon ending of epilepsy is in dementia, and that there are very few, if any, cases of epilepsy in which the shadows of this dementia are not forecast upon one or other of the mental faculties, especially upon the memory. Nor is it an objection to this view that men like Julius Cæsar, or Mahomet, or Napoleon I., should have had epileptic seizures; for who shall say, that the overwrought brains of these men may not have been broken down in some important particulars before these seizures made their appearance? And, certainly, there is no lack of evidence to show that the subjects of hysterical or chronic convulsion are in all respects the very reverse of "strong-minded."

87. *All signs of mental life are abolished, or on the point of being abolished, during the paroxysm of convulsion.*

In general, convulsion of an epileptic or epilepti-form character the mind is a perfect blank, and so also, with very few exceptions, in the partial forms of the same convulsions. In the convulsion of hysteria, and in the more severe forms of choreic movements, and the will is altogether in abeyance, and the intellectual state is one which approaches very closely to that of unconsciousness. Hence it is not to be supposed that convulsion is in any way connected with exalted functional activity of that part of the brain which ministers to the mental faculties.

88. *Convulsion must not be looked upon as a symptom of active inflammation of the brain or its membranes.*

In acute inflammation of the brain or its membranes, convulsion may occur during the cold stage of the disorder, or, more rarely, when the stage of true inflammation is subsiding into the stage of coma or collapse, but never during the existence of active phrenitis. It seems, indeed, to be a constant rule that the hot stage of active inflammation of the brain or its membranes is attended, not by convulsion, but by acute delirium, such as is met with in phrenitis. In the more passive forms of this inflammation, convulsion is a much more frequent phenomenon than in the acute forms, and it is not so easy to determine the relations of the convulsion to the inflammation; but, so far as I can make out, convulsion is altogether incompatible with the presence of the periods of vascular reaction, even though these periods be but faintly marked. In the great majority of these cases, indeed, true inflammatory excitement of the circulation is always absent; and the delirium which may be present is no proof to the contrary, for this delirium is of the type which is met with in delirium tremens, and not of the type which is met with in acute phrenitis. It would seem as if the cerebral disorder in these cases did not get beyond the stage of rigor,—the stage of "irritation," of which I shall have more to say presently. At any rate, I know of nothing in the clinical history of these passive forms of inflammation of the brain or its membranes which is at all calculated to contradict the previous conclusion, that convulsion is altogether incompatible with the period or periods of vascular reaction. Nor is there any evidence of a contrary character in the disclosures of the deadhouse.

89. *Convulsion must not be looked upon as a symptom of a congested condition of the cerebral veins.*

As it seems to me, the clinical history of disease is opposed to the theories which ascribe convulsion to a congested condition of the cerebral veins. In whooping-cough, these veins are often congested in a very high degree during the paroxysm, and yet convulsion is only an accidental accompaniment of the paroxysm. In congestion of the lungs, also, these veins are greatly gorged with black blood; and the consequence of this engorgement are dreamy sleepiness, stupor, perhaps, coma, rarely convulsion. Nor is the case different where extreme venous congestion of the brain is brought about by straining or in any other way; for here the symptoms are coma and paralysis, not coma and convulsion; apoplexy, not epilepsy. Moreover, the recent experiments of MM. Kussmaul and Tenner show very plainly that the effect of tying the internal and external jugulars of rabbits is *not* to convulse these animals, but only to stupify them for the first twenty-four or thirty hours, and, in some instances, to cause them to gnash their teeth for a short time. Indeed, there is nothing in all the evidence, physiological or clinical, to nullify the conclusion already drawn,—that venous blood has no special action in producing convulsion.

90. *The peculiar condition of the nervous system which is known under the name of "irritation," and which, in the majority of cases, has a great deal to do with the production of convulsion, is in no sense the equivalent of inflammation.*

The morbid condition of the nervous system, which is known under the name of "irritation" may be attended by various definite symptoms, and followed by various definite results. According as it may happen to affect the parts of the nervous system which minister to ordinary muscular movements, to common or special sensation, or to vascular movements, it may be attended by tremor, convulsion, or spasm, by morbid sensations of various kinds, or by a contracted condition of the vessels. According as it may happen to have affected one or other of the parts of the nervous system which have been named, it may be followed by paralysis of the ordinary muscular movements, by anæsthesia or some analogous condition of special sensation, or by congestion and inflammation. Moreover, it may be attended by partial or general delirium, and followed by partial or general stupor or coma, if its seat happen to be in the parts of the nervous system which minister to volition and reason. Nor is it difficult to see how it should be thus attended and thus followed.

The circumstances which give rise to the state of "irritation" in some part of the nervous system, and which favor the development of this state, are circumstances which appear to produce the reversal of the natural electrical relations of the exterior and interior of the nerve-fibres at the seat of "irritation." It has been seen in a former lecture that the natural electrical relations of the exterior and interior of the nerve-fibres are reversed when these fibres are acted upon by certain mechanical injuries or when their vitality is upon the point of extinction. It has been seen, that is to say, that the natural electrical relations of the exterior and interior of the nerve may be reversed under circumstances which may give rise to the state of "irritation" in a nerve or nerve-centre, and which favor the development of this state: for nothing is more certain than that mechanical injuries to a nerve or nerve-centre may give rise to a state of "irritation" in these parts, and that a depressed condition of the vital powers generally is favorable to the development of this morbid state. And thus there is no difficulty in supposing that the natural electrical relations of the exterior and interior of the nerve-fibres may be reversed at the seat of "irritation."

Now, in the case where the natural electrical relation of the exterior and interior of the nerve-fibres are preserved, all parts of the exterior of the fibres are electrified positively, and all parts of the interior negatively, and the electric condition of the exterior and interior appears to be one of static tension, so long as the nerve-fibre remains in a state of inaction. And this appears to be the case, because *all* parts of the exterior are electrified positively, and because *all* parts of the interior are electrified negatively; for it is a law of electricity that parts electrified with *similar* electricity repel each other. Nor is this condition of static tension *in* the exterior and *in* the interior of the nerve-fibres neutralized by a contrary action *between* the exterior and interior; for there is something in the constitution of the fibres, be that what it may, which keeps the exterior and interior in opposite electric conditions, and which prevents the opposite electricities of the exterior and interior from rushing together and neutralizing each other. But in the case where the natural electrical relations of the exterior and interior are reversed in a certain part of a nerve-fibre, instead of all parts of the exterior being electrified positively, and all parts of the interior negatively, some part of the exterior is electrified negatively, and some part of the interior positively, as is shown in the accompanying diagrams by making the parts of the fibre, which are occupied by negative electricity, of a darker shade than the parts which are occupied by positive electricity: and the result is that there must be a continual combination and disappearance of electricity *in* the exterior and *in* the interior between the parts of the fibre in which the natural electrical relations of the exterior and interior are preserved, and the part in which these relations are reversed,—between, that is, the parts which are indicated in the diagram by the letters AA, and the part which is indicated by the letter B. The result, that is to say, is one in which the nerve must lose electricity so long as this reversal continues; for it is a law of electricity that opposite electricities combine and neutralize each other if there be nothing to prevent them from yielding to their natural affinities. In other words, the result of the reversal must be to throw the nerve from the state of inaction into that of action; for it has been seen that a nerve loses electricity when it passes from a state of inaction into that of action, and that this loss is accompanied by the development of instantaneous electrical currents of high tension, analogous to the discharges of the torpedo in and near the nerve.

If, then, this be so,—if the natural electrical relations of the exterior and interior of the nerve-fibres are reversed at the seat of "irritation," and if the effect of this reversal is to throw the nerve from a state of inaction into that of action so long as the reversal continues,—then there is no difficulty in explaining the several phenomena which may attend upon or result from the morbid state of the nervous system which is known as "irritation." There is no difficulty in understanding how, according as its seat may be in ordinary motor nerves or ganglia, in sensory nerves or ganglia, in the cerebral hemispheres, or in the vaso-motor nerves or ganglia, the "irritation" may be attended by morbid contractions of the common muscles, by morbid sensations of various kinds, by morbid mental movements, or by morbid contractions of the bloodvessels; and how, according as its seat may have been in one or other of those parts of the nervous system which have just been mentioned, the "irritation" may end by paralyzing one or other of these parts, and by producing in this manner paralysis of the ordinary muscles, or anæsthesia and conditions allied to it, or partial and general stupor and coma, or congestion and inflammation.

According to this view, then, it is easy to see how, if the vaso-motor nerves or ganglia are implicated, the "irritation" may be attended by contraction of the vessels; for contraction of the vessels is produced when these nerves are acted upon by galvanism or in any other way. And it is as easy to see how, in the end, the same "irritation" may be followed by congestion and inflammation: for it has been seen that the contraction of the vessels passes into congestion and inflammation if the said nerves or ganglia be paralyzed either by the continuance of the same cause which first put them in action, or in any other way. (Indeed, the experiments of Professor Claude-Bernard and Dr. Brown-Séquard upon the sympathetic in the neck appear to furnish the key to the interpretation of inflammation when it is used in this manner.) Nay, it is easy to see how this congestion or inflammation may be seated anywhere or everywhere; for there is no part of the nervous system in which the "irritation" may not begin, or into which it may not travel, and therefore there is no part of the vaso-motor system which may not be paralyzed by the continuance of the "irritation,"—paralyzed, that is, by the continuance of the action of those instantaneous electrical currents of high tension, analogous to the discharges of the torpedo, which are developed in and near the nerve or nerve-centre, which is the seat of "irritation."

According to this view, then, "irritation" is in no sense the equivalent of inflammation. According to this view, indeed, the *hot stage* of inflammation, with its congested and inflamed vessels, *must follow the cold stage*, with its rigors and pains, and with its shrunken and contracted vessels, because the hot stage is not produced until the "irritation," which produces at one and the same time and by one and the same means the rigors and pains and empty vessels of the cold stage, has by its continuance at last brought about a certain amount of paralysis in the nerves belonging specially to the vessels. And certainly there is nothing in the history of convulsion which is contradictory to this view of the relations between "irritation" and inflammation. For is it not true that the epilepsy may be present in its most violent forms where it is impossible to associate the "irritation" upon which the convulsion depends, with the faintest blush of inflammation in any part of the nervous system or elsewhere? And has it not just been seen that the epileptiform convulsion which may be associated with cerebral inflammation is anterior or posterior to this inflammation,—is, in fact, a substitute for rigor or subsultus?

91. *The condition of the function of innervation during convulsion is one which supports the notion, that the convulsion is connected with depressed and not with exalted action in the function.*

All the previous considerations lead to this conclusion, and to this conclusion only.

92. *The condition of the circulation and respiration during convulsion is one which necessitates the conclusion that convulsion is connected with depressed and not with exalted action of the nervous system.*

This conclusion is also inevitable, for it has been seen that the condition of the circulation and respiration during convulsion is incompatible with anything but the most depressed condition of vital action in the nervous system and in the system generally.

93. THE GENERAL CONCLUSION TO BE DEDUCED FROM THE CONSIDERATION OF THE CONDITION OF THE RESPIRATION, AND CIRCULATION, AND INNERVATION, DURING CONVULSION, IS THIS, —THAT THE PATHOLOGY OF CONVULSION IS AS MUCH IN HARMONY WITH THE VIEW OF MUSCULAR MOTION SET FORTH IN THESE LECTURES AS IT IS OUT OF HARMONY WITH THE CURRENT VIEW ON THIS SUBJECT,—THAT, IN FACT, CONVULSION IS THE SIGN OF DEPRESSED, AND NOT OF EXALTED, VITAL ACTION.

### LECTURE VI.

### 2. ON THE THERAPEUTICS OF CONVULSION.

I spoke at some length upon this subject in the Gulstonian Lectures, which I had the honor of delivering in this College three years ago; and I do not propose to trespass upon your patience by recapitulating what I then said. Indeed, all I propose to do at present is to offer a few detached hints upon some points which seem to be of primary importance, and which have some claim to novelty.

94. *There is reason to believe that the diet in many cases of chronic convulsive disorder ought to contain somewhat more than an average quantity of oily and fatty matters, and somewhat less than an average quantity of lean meat.*

There is a common notion, not confined to non-medical persons, that lean butcher's meat is the one thing necessary to strengthen a weak system; and I believe that the carrying out of this notion not unfrequently complicates the difficulties which prevent the successful treatment of the case under consideration, and of many other cases also. With such a diet, as it seems to me, the blood is likely to get into a semi-gouty condition, unless there be a degree of activity in the circulation and respiration which is not likely to be met with in epilepsy, chorea, hysteria, or any other form of chronic convulsive disorder; and I have an impression that the undoubtedly beneficial influence of bromide of potassium in so many cases of epilepsy is owing, in part at least, to the fact that this salt, like iodide of potassium, corrects the semi-gouty condition of the blood arising from this and other causes. At any rate, I have no doubt as to the practical advantages of so regulating the diet in the cases under consideration as to diminish the usual allowance of fibrinous articles and to increase the usual allowance of oily and fatty articles. I have increased the quantity of the latter articles for the same reason as that which has led me to employ cod-liver oil in many of these cases, and of this I shall have to speak presently.

95. *There is reason to believe that gymnastic exercises are very beneficial in the great majority of chronic convulsive cases.*

During the last three or four years, I have seen several cases of epilepsy, chorea, and hysteria, in which undoubted good has resulted from the adoption of a regular course of suitable gymnastic exercises; and the more I see the more I am satisfied that a course of this kind is a very important adjuvant in the treatment of these and many other cases. I recall to mind more than one case of epilepsy in which the patient has said, that he has often warded off an attack which seemed to be imminent by a bout at the trapezium; and I have at present a case under treatment in which good seems to have been done by adopting a practice recommended by Dr. Henry Silvester, in the treatment of consumption,—a practice which may, perhaps, be brought under the head of gymnastics. Having ascertained that the mere dead weight of the arms hanging by the side reduces the amount of air which might be taken into the chest, to the extent of ten cubic inches or thereabouts, Dr. Silvester proposes that a phthisical person shall now and then eke out his insufficient respiration by breathing in such a manner as to get rid of this weight,—by breathing, that is to say, with the hands taking hold of something fixed at a sufficiently high level, or, what answers the purpose still more easily, with the hands clasped together and resting upon the top of the head. And this proposition appears to have much to recommend it, not only in phthisis, but also in other cases in which, as in epilepsy, the respiration is wanting in activity. I also think that a collateral argument in favor of gymnastics may be derived from Dr. Silvester's investigations upon artificial respiration, for these investigations show that as much as from nine to forty-four cubic inches of air may be made to pass in and out of the chest by merely pulling the arms upwards and then bringing them backwards to the sides, and that this movement of the arms is itself a mode of performing artificial respiration which is more effectual than any other. Of course, the beneficial influence of gymnastics is not confined to the respiratory function. On the contrary, this influence tells equally upon the circulatory and upon all other functions, as it indeed must do if it acts in this manner upon the respiration, for the activity of the respiration is a fair criterion of vital action generally.

96. *There is reason to believe that the action of cod-liver oil is beneficial in the great majority of cases of chronic convulsive disorder.*

For the last four years I have employed cod-liver oil in many cases of epilepsy, chronic epileptiform disorder, chorea, and hysteria; and, so far as I can judge, I have no reason to be dissatisfied with the results. I can also refer to the experience of my friend and colleague, Dr. Anstie, as bearing out my own experience in this respect most fully. I was led to this practice, and also to that of recommending a fair amount of fatty and oily articles of diet, by remembering that fatty matter is, as is seen in the following table, (which gives the mean of six

| | Infants. | Youths. | Adults. | Aged persons. | Idiots. |
|---|---|---|---|---|---|
| Fat, | 3·45 | 5·30 | 6·10 | 5·32 | 5· |
| Phosphorus, | ·80 | 1·65 | 1·80 | 1·09 | 0·85 |
| Albumen, | 7· | 10·20 | 9·40 | 8·65 | 8·40 |
| Osmazone and Salts, | 5·96 | 8·59 | 10·19 | 12·18 | 14·82 |
| Water, | 82·79 | 74·26 | 72·51 | 73·65 | 70·93 |

analysis of the human brain, by M. L'Heretier,) as important ingredient of brain-tissue; and not of brain-tissue only, for it is found that the ingredients of the spinal cord and of the nerves generally are substantially the same. Remembering this fact, and remembering also the reasons which oblige me to believe that the function of innervation is carried on imperfectly in all convulsive maladies, I came to the conclusion that there might be a starved condition of the nerve-tissue, which might, perhaps, be remedied by cod-liver oil or by oily and fatty articles of food; and, be this theory right or wrong, I think, as I have said, that I have no reason to be dissatisfied with the results of putting it in practice.

97. *There is reason to believe that phosphorus is a suitable remedy in many cases of chronic convulsive disorder.*

For the last four years, I have used phosphorus in the majority of the cases of convulsion in which I have used cod-liver oil, and for the same reasons, and with the same results. I asked myself whether the fact set forth in the preceding table, (¶ 96,) that phosphorus is present in nerve-tissue, and that the amount of this ingredient seems to have some direct relation to the activity of the nervous function, being as much as two per cent in adult life, and below one per cent in infants and idiots, might not show that phosphorus is required as food by a weak nervous system,—as much required, perhaps, as is iron in cases where there is a deficiency of red corpuscles in the blood; and this question, once put, seemed to require an answer in the affirmative. "In small doses," says Dr. Pereira, "phosphorus excites the nervous, vascular, and secretary organs. It creates an agreeable feeling of warmth in the epigastrium, increases the fulness and frequency of the pulse, augments the heat of the skin, heightens the mental activity and the muscular powers, and operates as a powerful sudorific and diuretic." In large doses, phosphorus, without doubt, is a caustic poison; in proper doses it produces the very changes which are necessary in epilepsy and in other cases of chronic convulsive disorder. In proper doses, and under the eye of a medical man, it is quite innocent of harm, and it may be productive of much good. This inference is that which may be drawn from what I said; and this inference, so far as I can see, is not contradicted by experience. Given in the large doses in which phosphorus has been given in a few cases already on record, the good resulting may have been doubtful, very doubtful; but this experience is nothing to the point, for there is no reasoning in any case as to the effects of medicinal doses from the effects of poisonous doses. Given in medicinal doses, I have seen enough to know that this remedy may be given, not only without harm, but with the unmistakable promise of real and substantial good. The form in which I first gave the phosphorus was the phosphorated oil of the Prussian Pharmacopœia, a preparation which is made by dissolving twelve grains of phosphorus in a fluid ounce of almond oil, by the aid of warm water. About four grains of the phosphorus is taken up, and the usual dose is from five to ten minims. I gave this oil always with cod-liver oil, in a little orange wine, twice or thrice a day. In many cases, however, this mixture proved to be so nauseous that the stomach refused to tolerate it; and lately I have often given the oil and the phosphorus separately, using the ethereal tincture of phosphorus of the French Codex as the vehicle for the phosphorus. I have given the oil with tolerable regularity so long as it seemed to be wanted, and the tincture now and then, especially when the symptoms called for a stimulant. I direct one fluid drachm of the ethereal tincture to be mixed with two fluid ounces of sulphuric ether, and preserved in a capped bottle, the dose being half a fluid drachm to one fluid drachm, mixed with water at the instant of swallowing it. In the ethereal tincture of phosphorus of the French Codex, four grains of phosphorus are dissolved in one fluid ounce of ether, and, consequently, the strength is the same as that of the phosphorated oil of the Prussian Pharmacopœia.

98. *There is reason to doubt the suitableness of belladonna as a remedy in many cases of epilepsy and other forms of chronic convulsive disorder.*

I have for some time been in the habit of looking upon the dilatation of the pupil, which results from the action of belladonna, as an objection to the employment of this medicine in epilepsy, and in cases more or less akin to epilepsy; and my chief reason for so doing has been a growing impression that the alterations in the size of the pupil are due to vascular rather than to muscular changes in the iris, and that these alterations furnish an exact index to the condition of the circulation within the brain. I have, in fact, been led to agree with those physiologists who look upon contraction of the pupil as being, in a great measure, due to a state of turgescence in the vessels of the iris, and upon dilatation of the pupil as being to the same extent the consequence of an empty state of those vessels. I have been led to do this, partly by thinking, as did Bichât that the pupil enlarges in the dark, not because there is then an end to the "irritation" which had kept certain sphincter fibres in a state of contraction so long as they are acted upon by the light, but because the *iris* has passed out of a state of expansion, which state was determined by the action of the light,—a view which brings the movements of the iris in the light and the movements of the cushions of the sensitive plant under similar circumstances into the same category; and partly by reflecting upon the history of five cases of acute cerebral meningitis which have been under my care during the last eighteen months. In these cases I saw the pupil contracted to its utmost limit when the symptoms denoted unequivocal active determination of blood to the head, and dilated before this state was developed and after it had passed off: and the more I pondered upon these phenomena, the more I became convinced that the size of the pupil was dependant upon the condition as to fulness or emptiness of the minute arteries and capillaries of the brain; that, in fact, the pupil became contracted because the vessels of the iris participated in the arterial turgescence of the brain, and that the pupil became dilated because the vessels of the iris participated in the emptiness of the cerebral vessels. And I was also confirmed in this opinion by remembering that the pupil contracts when the eye and the whole of the corresponding side of the head become injected with blood as the result of dividing the cervical sympathetic in that side, and that the pupil dilates when this vascular injection is made to pass off by galvanizing the divided sympathetic above the line of section. In the dilatation of the pupil, resulting from the action of belladonna, therefore, I have had what seemed to me a plain proof that this remedy produced an anæmic condition of the brain, and an equally plain objection to the use of this remedy in epilepsy and in any other cerebral disorder in which there is reason to believe that the brain and nervous system generally are in an anæmic rather than in a hyperæmic condition; and I can say, without any hesitation, that I have seen no such unequivocal good from the practical employment of belladonna in these cases as to lead me to disregard this theoretical objection. Upon this point, however, I may not have seen enough to enable me to form a sound conclusion.

99. *There is reason to believe that opium may be a more suitable remedy than belladonna in some cases of epilepsy and in some other forms of convulsive disorder.*

Opium differs from belladonna in causing contraction of the pupil instead of dilatation; and, therefore, for the reasons set forth in the last paragraph, opium differs from belladonna in producing a hyperæmic instead of an anæmic condition of the brain, and in being suitable in cases in which belladonna is not suitable,—in cases where the brain is anæmic rather than hyperæmic,—in cases, it may be, of epilepsy, among others. As yet, however, I have met with few cases in which I have thought it expedient to test the correctness of this theory by putting it in practice.

100. *There is reason to doubt the efficacy of zinc as a remedy in epilepsy, and in cases akin to epilepsy.*

I am disposed to look upon zinc as having an action upon the system which is directly opposed to that of iron. Iron, as all know, has an astonishing power of favoring the nutrition and multiplication of the blood-corpuscles. Zinc, on the other hand, blanches the system, and induces, before long, the state which was once known as *tabes sicca*, and for which no other name has yet been devised. Zinc, indeed, exercises a peculiar desiccating influence upon the system. Dr. Pereira mentions the case of an epileptic gentleman who took daily, upon an average, twenty grains of oxide of zinc, until he had swallowed 3246 grains, and who at the end of this time was pale in the extreme, sallow, wasted away, almost idiotical, with the tongue thickly coated, the bowels constipated, the inferior limbs cold and œdematous, the abdomen tumid, the arms cold and shrivelled, the skin dry and almost like parchment, the pulse slow, thready, and scarcely perceptible. This patient experienced no change for the better in his fits; but he soon recovered from the effects of the zinc under appropriate treatment. I have also myself seen four cases, different from this one only in being not quite so severe, and I have met with many cases in which the prolonged use of zinc, in one form or another, has produced decided sallowness and bloodlessness of the complexion. I find also that brass-founders, who are exposed to the fumes of deflagrating zinc, are often dried up and wizened in a curious manner. And Dr. Greenhow has recently shown, in addition, that these men are apt to suffer, particularly in the afternoons of the days spent in the casting shop, from what is called "brass-founders' ague,"—a disease beginning with malaise, tightness or constriction in the chest, nausea, repeated rigors, and ending in profuse sweating after a short and faintly marked hot stage. Here, then, is evidence that zinc is capable of producing a form of convulsive malady,—for rigor is a form of such malady,—as well as of producing the *tabes sicca* which has been described. Here, indeed, is evidence which may, perhaps, throw some light upon the disorders of the nervous system, in which zinc is likely to do good or harm. That zinc does not always do good in these disorders is evident. My colleague, Dr. Marcet, who has for some time past given oxide of zinc very extensively in these disorders, reports very favorably of the result in some cases, but not in others. He does not report favorably of this mode of treatment in ordinary cases of epilepsy, and his report, I am satisfied, will not clash with the experience of the great majority of practitioners. Nay, it is a significant fact that M. Herpin, who has written a thick volume in praise of the virtues of oxide of zinc in epilepsy, has for some time been dissatisfied with his own favorite remedy, even to the extent of discarding it not unfrequently for a remedy which savors more strongly of the *materia medica* of the middle ages than that of the nineteenth century—poudre de Neufchâtel, or, in plain words, powder of fried mole. Dr. Marcet reports more favorably of the action of ozide of zinc in those cases in which he had to deal with various vague head symptoms, without convulsion,—a result which will also coincide with the experience of not a few. How then is this? Is it that the zinc does good in those cases of brain disorder in which there is a disposition to congestion of the brain, without any deficiency in the amount and quality of the blood, by virtue of that power which produces *tabes sicca* when pushed to an injurious extent? Is it that the zinc does good when the condition to be combated is hyperæmic, and harm when this condition is anæmic? These are questions to which I know no better answers than that which is contained in the preceding considerations, and this answer is so plain as to require no further comment to make it plainer.

101. *There is reason to believe that alcoholic stimulants are the most trustworthy antispasmodics in the prevention and treatment of convulsion.*

The wider experience of the last four years has not shaken my convictions upon this point. On the contrary, I do not remember any one case in which there was not something to strengthen these convictions. I have very recently seen a case of aggravated chorea in which there had been no sleep for five days and nights, and no cessation to the movements of any moment, in which a wineglass of port wine given every half-hour, with an egg beaten up in the alternate dose, produced quiet and sleep in ten hours, and in which a continuance of the same treatment, only in a less vigorous style, left the patient well, so far as the chorea was concerned, in a week; and I could cite other cases, at least three, to the same effect. I could also cite many cases of epileptic and hysteric convulsion averted, or its recurrence prevented, by means of a proper use of alcoholic stimulants.

102. *There is reason to believe that bloodletting, in one form or another, may be permitted in certain cases of convulsion, in order to prevent certain consequences of the convulsion.*

There is nothing in the pathology of convulsion to justify the notion that convulsion is likely to be prevented by bloodletting; but it is not difficult to understand that cases of epileptic or epileptiform convulsion may be met with in which the veins of the brain may be so gorged with black blood as to put the patient in imminent danger of apoplexy, and in which this danger may be somewhat lessened by abstracting a small quantity of blood. And, possibly, this may be no wrong practice in such a case. At present it is plain that past experience is too much disregarded in this matter. Why, it may well be asked, if moderate bloodletting be so serious a matter, should a woman require periodical bleeding to keep her in health? Nor must the astonishing power of multiplication belonging to the blood-corpuscles be lost sight of in this matter. Speaking of the rapidity with which an anæmic patient became convalescent in St. Mary's Hospital, Dr. Thos. King Chambers says,—and what he says requires no further comment,—"She weighs 8 stone, or 1792 oz.: of this 2-7, or 512 oz., is blood; and of this blood 133-1000, or 60 oz., should be red globules. Now, the analysis of MM. Andral and Gavarret show that in cases of anæmia of at all a marked character, (as this was,) we may expect at least three-fourths of the red blood-discs to disappear; so that when she came into the hospital it may fairly be assumed that she did not possess more than 15 oz.; and now I think she may be assumed with equal fairness to have got up to 45 oz., which is conceding that she still wants a-quarter of perfect health. By this she must have made 20 oz. of red blood-discs, —that is the most important organic constitutent of upwards of 150 oz. of blood,—in a month.

103. THERAPEUTICALLY AS WELL AS PATHOLOGICALLY, THERE IS, IN FACT, EVERY REASON TO BELIEVE THAT THE MEANS TO BE EMPLOYED IN THE TREATMENT OF CONVULSION ARE THOSE WHICH EXALT, AND NOT THOSE WHICH DEPRESS, VITAL ACTION.

